# Pharmacogenetics of Risperidone and Cardiovascular Risk in Children and Adolescents

**DOI:** 10.1155/2016/5872423

**Published:** 2016-01-06

**Authors:** Amilton Dos Santos-Júnior, Taciane Barbosa Henriques, Maricilda Palandi de Mello, Osmar Henrique Della Torre, Lúcia Arisaka Paes, Adriana Perez Ferreira-Neto, Letícia Esposito Sewaybricker, Thiago Salum Fontana, Eloisa Helena Rubello Valler Celeri, Gil Guerra-Júnior, Paulo Dalgalarrondo

**Affiliations:** ^1^Department of Psychiatry, School of Medical Sciences (FCM), State University of Campinas (Unicamp), 13083-887 Campinas, SP, Brazil; ^2^Laboratory of Human Genetics, Center for Molecular Biology and Genetic Engineering (CBMEG), Unicamp, 13083-875 Campinas, SP, Brazil; ^3^Growth and Development Laboratory, Center for Investigation in Pediatrics (CIPED), FCM-Unicamp, 13083-887 Campinas, SP, Brazil; ^4^Department of Pediatrics, Pediatric Endocrinology Unit, FCM-Unicamp, 13083-887 Campinas, SP, Brazil

## Abstract

*Objective*. To identify the frequency of obesity and metabolic complications in child and adolescent users of risperidone. Potential associations with clinical parameters and SNPs of the* HTR2C*,* DRD2*,* LEP*,* LEPR*,* MC4R*, and* CYP2D6* genes were analyzed.* Methods*. Samples from 120 risperidone users (8–20 years old) were collected and SNPs were analyzed, alongside assessment of chronological and bone ages, prescribed and weight-adjusted doses, use of other psychotropic drugs, waist circumference, BMI *z*-scores, blood pressure, HOMA-IR index, fasting levels of serum glucose, insulin, cholesterol, triglycerides, transaminases, and leptin.* Results*. Thirty-two (26.7%) patients were overweight and 5 (4.2%) obese. Hypertension was recorded in 8 patients (6.7%), metabolic syndrome in 6 (5%), and increased waist circumference in 20 (16.7%). The HOMA-IR was high for 22 patients (18.3%), while total cholesterol and triglycerides were high in 20 (16.7%) and 41 (34.2%) patients, respectively. SNP associations were found for* LEP*,* HTR2C*, and* CYP2D6* with BMI;* CYP2D6* with blood pressure, ALT, and HOMA-IR;* HTR2C* and* LEPR* with leptin levels;* MC4R* and* DRD2* with HOMA-IR;* HTR2C* with WC; and* LEP* with ALT.* Conclusions*. Although not higher than in the general pediatric population, a high frequency of patients was overweight/obese, with abnormalities in metabolic parameters and some pharmacogenetic associations.

## 1. Introduction

Risperidone, a serotonin-dopamine antagonist, is effective in preventing delusions and hallucinations in addition to impulsive and aggressive behavior [1, 2]. The US Food and Drug Administration has approved the use of risperidone in children to treat irritability related to autism (5–16 years old), manic or mixed state in bipolar disorder (10–17 years old), and schizophrenia (13–17 years old) [3]. However, the off-label use of risperidone to treat a series of other behavioral symptoms including developmental and disruptive disorders is not uncommon [4].

Although widely prescribed, risperidone use has some risks and few studies have evaluated its pharmacokinetic effects in children and adolescents or adverse drug reactions in these populations [4]. The possibility of weight gain, cardiovascular effects, and metabolic effects associated with hepatotoxicity, insulin and leptin resistance, hyperglycemia, and hyperinsulinemia is associated with increased cardiovascular and cancer morbimortality in adulthood [5, 6].

Regarding the weight gain and metabolic effects induced by risperidone, the* HTR2C*,* DRD2*,* LEP*,* LEPR*,* MC4R,* and* CYP2D6* genes are believed to be involved. Considering that serotonin receptors directly influence the satiety and appetite control systems [6], the X-linked* HTR2C* gene is likely to play an important role as it encodes the serotonin 2C receptor [7].

Another gene involved in weight gain is the* DRD2* gene, which encodes the dopamine D2 receptor [8] and is associated with obesity [9]. It has been reported that the availability of striatal dopamine D2 receptors is significantly reduced in overweight individuals [9].

The* LEP* and* LEPR* genes, which encode leptin and its receptor, respectively, have also been reported to be important in antipsychotic-induced weight gain studies [10]. Secreted only by the adipose tissue, leptin inhibits food intake and increases energy expenditure [3, 11].

Melanocortin-4 Receptor (*MC4R*) is a transmembrane G protein-coupled receptor expressed in the central nervous system, primarily in the hypothalamus [11, 12].* MC4R* plays a critical role in the regulation of energy homeostasis by leptin, and heterozygous mutations in the gene account for 0.5–6.3% of severe or early-onset obesity cases, which represent the most frequent cause of monogenic human obesity syndromes [6].

The gene coding the P450 2D6 enzyme (*CYP2D6*) is one of the best studied pharmacogenes [13]. Approximately 25% of all drugs, including risperidone, are metabolized through this enzyme, which also happens to be one of the most polymorphic enzymes with over 100 known allelic variants [13]. It is now known that polymorphisms in the* CYP2D6* gene can result in a range of effects from complete loss of enzyme activity through deletion of the gene (poor metabolizer status), or extensive activity, through duplication of the gene (ultra-rapid metabolizer status) [13]. Besides the involvement of other genes in the pharmacodynamics of risperidone, pharmacokinetic studies have hypothesized that low activity of the cytochrome complex may be associated with an increase in serum risperidone levels, which can indirectly lead to weight gain [1, 5, 14].

The aims of this study were to assess the frequency of obesity, hypertension, metabolic syndrome, insulin resistance, and dyslipidemia in a sample of child and adolescent users of risperidone, to evaluate associations with clinical and pharmacological data and with certain polymorphisms of the* HTR2C*,* DRD2*,* LEP*,* LEPR*,* MC4R*, and* CYP2D6* genes.

## 2. Subjects and Methods

### 2.1. Study Design and Participants

This was a cross-sectional study, with a sample composed of patients attending the psychiatric outpatient service of the hospital of the University of Campinas (Unicamp) aged 8–20 years old and undergoing treatment with risperidone for mental and behavioral disorders. Inclusion criteria were (a) not being overweight or obese (BMI less than +1 standard deviation (SD) for age and sex) and not having any other illness or use of drugs known to predispose to obesity or metabolic syndrome at the beginning of the study; (b) not having mental or behavioral disorders due to physical ailments; (c) not being a user of psychoactive substances; (d) not having been diagnosed with severe intellectual disability; (e) no diagnosis of an eating disorder; and (f) in the case of women, not pregnant, nursing, or using hormonal contraception. Only patients who freely gave informed consent (or their guardians) were included in the study.

### 2.2. Methods

#### 2.2.1. Clinical Evaluation

The following data were evaluated: gender (male or female), chronological age, bone age (TW2 20-bone method) [15], period of time of treatment with risperidone (24 months before the study at the most), current dose of risperidone, use of other psychoactive drugs, weight (kg, measured with a calibrated digital scale balance), height (m, measured with a stadiometer), waist circumference (cm, measured at the level of the umbilical scar), and systolic and diastolic blood pressure (BP in mmHg, measured with pediatric cuffs and with values adjusted for the arm diameters). The BMI (kg/m^2^) was calculated and transformed into a *z*-score [16]. BMI *z*-scores, also called BMI standard deviation scores, are clinical measures of relative weight adjusted for a child or an adolescent age and sex [17]. Given its age, sex, BMI, and an appropriate reference standard, a BMI *z*-score (or its equivalent BMI-for-age percentile) can be determined [17]. In this study, the external reference used was the one established by the WHO [16, 17]. Obese patients were defined as having *z*-scores of at least +2 SD and overweight patients as having a *z*-score of at least +1 SD but less than +2 SD [16]. A high waist circumference was defined as ≥90th percentile for age, sex, and ethnicity [18]. Hypertension was defined following the National Heart, Lung and Blood Institute criteria, correlated with percentiles specific to sex, height, and age [19].

#### 2.2.2. Laboratory Evaluation

Serum glucose, total cholesterol, LDL, HDL, triglycerides (enzymatic colorimetric method), insulin (immunofluorometric assay), aspartate aminotransferase (AST), alanine transaminase (ALT) (ultraviolet optimized kinetic method), leptin (enzyme-linked immunosorbent assay), thyroid-stimulating hormone (TSH) (chemiluminescence), and free thyroxin (T4) (chemiluminescence) levels were assessed in the Human Physiology Laboratory of University of Campinas clinical hospital after 12 hours of fasting. Thyroid hormone levels were measured to exclude patients with hypo- or hyperthyroidism.

The relationship between insulin and fasting glucose levels was used to calculate the Homeostatic Model Assessment of Insulin Resistance (HOMA-IR), which is considered normal when ≤2.89 [20]. The normal levels for serum lipids were triglycerides <75 mg/dL in children up to nine years of age, and <90 mg/dL from ten years onwards; HDL cholesterol >45 mg/dL; LDL cholesterol <110 mg/dL; and total cholesterol <170 mg/dL [21]. The criteria for the diagnosis of diabetes mellitus were fasting blood glucose level ≥126 mg/dL [22]. The diagnosis of metabolic syndrome was based upon the International Diabetes Federation criteria, including both clinical and laboratory parameters: abdominal obesity (waist circumference ≥90th percentile for patients under 16 years old and, for those aged 16 years old or more, waist circumference ≥94 cm for males or ≥80 cm for females) and two or more of the other four demanded indexes (triglycerides ≥150 mg/dL; HDL-cholesterol <40 mg/dL; blood pressure ≥130/85; and fasting glycemia ≥100 mg/dL): [23].

Genomic DNA was extracted from total venous blood by proteinase K lysis (Boehringer Mannheim, Germany). The following single nucleotide polymorphisms (SNPs) were determined in the Human Genetics Laboratory of the Molecular Biology and Genetic Engineering Center, Unicamp, by real-time polymerase chain reaction (PCR), according to the* TaqMan* allelic discrimination assay (Applied Biosystems, Foster City, CA, USA):* HTR2C*: SNP rs6318 (c.68G>C; p.Cys23Ser) and rs3813929 (c.-759C>T);* LEP*: SNP rs7799039 (-2548A>G);* LEPR*: SNP rs1137101 (c.668A>G, p.Gln223Arg);* DRD2*: SNP rs1799978 (c.-241A>G) and rs6277 (C957T);* MC4R*: SNP rs17782313 (3′ region of the gene);* CYP2D6*: SNP rs72552269 (c.100C>T, p.Pro34Ser, allele* CYP2D6∗10*).

The amplification reactions were performed in a 7500 Fast Real-Time PCR System (Applied Biosystems, Foster City, CA, USA). The total volume of all reactions was 7 *μ*L, containing* TaqMan Genotyping PCR Master Mix 2x* (3.5 *μ*L), SNV* Genotyping Assay* 40x (0.175 *μ*L), MiliQ water (2.325 *μ*L), and 10 ng of each genomic DNA (1 *μ*L). Data were recorded and analyzed using 7500 System Sequence Detection Software© (SDS). The reactions were performed in 96-well optical plates (0.1 mL MicroAmp, Applied Biosystems, Foster City, CA, USA) and submitted to the following temperatures and cycles: 10 min denaturation at 95°C, 40 cycles of 15 seconds at 95°C, and 1 min extension at 60°C.

*Ancestry Characteristics of the Patients*. Racial miscegenation is highly prominent in Brazil because Europeans of white skin color, Africans of black skin color, Asians of yellow skin color, and indigenous peoples of Brazil participated in the process of the formation of this country [24]. The mixing has been intense since the beginning of colonization [24]. The small number of white women among the Portuguese colonizers led the latter to have relationships with native indigenous and black slaves [24]. This mixing gave rise to other groups such as “mulattos” (the mix of white and black), “caboclo,” or “mameluco” (the mix of white and indigenous peoples), and the “cafuzo” (the mix of black and indigenous peoples) [24]. Pena et al. showed recently that in Brazil one cannot predict the color of persons from their genomic ancestry [24].

As a result, miscegenation in Brazil is an aspect that must always be considered in any study using racial analysis [25]. The analysis adopted by Instituto Brasileiro de Geografia e Estatistica (IBGE) has been considered as official since 1991 for racial classification in demographic studies [25]. This classification uses self-declaration in the collection of data, that is, the person chooses from a list of five racial categories (white, black, mulatto, yellow, and indigenous peoples) [25]. The IBGE defines individuals who declare themselves as mulatto or black as “black” [25].

### 2.3. Statistical Analysis

Statistical analysis was carried out using SPSS version 22 (IBM Co., Armonk, NY, USA). Chi-square or Fisher's exact test was used to assess the presence or absence of obesity, WC increase, hypertension, HOMA-IR changes and triglyceride concentrations, and cholesterol (total and fractions), in relation to sex, and any combination of these with the concomitant use of other psychoactive drugs, compared to that found in each SNP. To assess correlations between age, BMI, BP, prescribed dose of risperidone, medication usage time, and the continuous distribution of routine laboratory assessments, the Spearman correlation coefficient was used. To compare clinical and laboratory biochemical and physiological parameters in relation to the studied SNPs and nutritional status, the Kruskal-Wallis and Mann-Whitney tests were applied. Significance was considered to be *p* = 0.05.

## 3. Results

The sample consisted of 120 participants (81.7% males) with a mean age of 13 ± 3.1 years. The majority had an externalizing disorder and received 0.04 ± 0.03 mg/kg/day of risperidone. Among the biochemical and hormonal parameters, only AST and leptin levels were statistically different when analyzed by gender: AST levels were greater in males (*p* = 0.009; Mann-Whitney test) while leptin was higher in females (*p* = 0.0001; Mann-Whitney test).

Risperidone monotherapy was being used by 32 (26.7%) patients. In 49 (40.9%) patients, another psychotropic drug was being used in combination with risperidone; two psychotropic drugs in 30 (25%) patients; and three or more psychiatric drugs in 9 (7.5%) patients. The psychiatric drugs used in association with risperidone were antidepressants in 53 (60.2%) patients; psychostimulants in 27 (30.7%); clonidine in 19 patients (21.6%); anticonvulsants in 11 (12.5%); lithium carbonate in 6 (6.8%); benzodiazepines in 8 (9.1%); nonbenzodiazepine sedative drugs (promethazine, methotrimeprazine, or pericyazine) in 7 (8%); other atypical antipsychotics in 6 (6.8%); and biperiden in 1 patient (1.1%).

There was no statistically significant difference in the occurrence of obesity, altered WC, hypertension, or metabolic syndrome in relation to sex, or whether or not the user was receiving risperidone monotherapy (Table 1). Metabolic syndrome was present in one (20%) obese case and in five (15.6%) of overweight cases. No patient had hypo- or hyperthyroidism.

There was no difference between the sexes regarding values recorded for HOMA-IR index (*p* = 0.122, Fisher's exact test), increased LDL cholesterol (*χ* ^2^ = 2.187; *p* = 0.139), total cholesterol increase (*χ* ^2^ = 1.295; *p* = 0.255), HDL cholesterol reduction (*χ* ^2^ = 0.028; *p* = 0.868), or increased triglycerides (*χ* ^2^ = 1.526; *p* = 0.217). There was no statistical difference between patients receiving risperidone monotherapy or risperidone in combination with other drugs in relation to HOMA-IR index (*χ* ^2^ = 2.339; *p* = 0.126), triglyceride levels (*χ* ^2^ = 0.001; *p* = 0.977), total cholesterol (*χ* ^2^ = 0.512; *p* = 0.474), HDL (*χ* ^2^ = 1.364; *p* = 0.243), and LDL (*χ* ^2^ = 0.017; *p* = 0.898).

Clinical and laboratory parameters and nutritional status are shown in Table 2. There was a correlation between the dose of risperidone with dose per kilogram of body weight (*r* = 0.850, *p* = 0.0001), chronological age (*r* = 0.260; *p* = 0.004), bone age (*r* = 0.305; *p* = 0.001), and medication usage time (*r* = 0.357; *p* = 0.0001). There was positive correlation between age and WC, SBP, and DBP, and a negative correlation with AST. Positive correlation was observed between bone age and WC, SBP, DBP, HOMA-IR index, and negative correlation between bone age and HDL and AST. Positive correlations were also seen between dose of risperidone and SBP, and DBP (Table 3), with negative correlation between the risperidone dose per kilogram of body mass and leptin. There were no significant correlations between duration of use of risperidone and the clinical and laboratory parameters evaluated (Table 3). Categorical variables related to clinical and laboratory changes, evaluated in relation to the distribution of the alleles of the evaluated SNPs, are shown in Table 4, while comparisons for continuous variables are shown in Table 5.

## 4. Discussion

The study revealed that the leptin concentrations (Table 4) had significant genetic associations: heterozygotes (C/T) for SNP rs3813929 in the* HTR2C* gene had higher serum leptin concentrations than hemizygous C/- or T/- (in males) or homozygous (C/C or T/T in female patients). This association, however, was not sustained when the analysis was separated by gender. McCarthy et al. have previously reported an association between leptin concentrations and a different SNP of the* HTR2C* gene [26].

In this study, SNP rs6318 from the* HTR2C* gene was associated with not being eutrophic when risperidone was being used, which was more frequent in carriers of the G allele, although this association was not maintained when the analysis was separated by gender. In female patients, the condition of being heterozygous (C/L) was associated with an increased WC when compared with homozygotes (C/C or G/G). Lett et al. suggest that, since previous negative studies related to this gene have involved long periods of risperidone usage, it is likely that the* HTR2C* gene is involved with adverse effects that occur at the start of antipsychotic therapy [5]. This is consistent with the findings of our own study, where the average time of drug use was more than 1.5 years [5].

Leptin has beneficial effects on glucose metabolism, acting on the hypothalamus with an antidiabetic effect, and its increased concentration suggests a resistance to its effects [22]. Its association with the* HTR2C* gene may be linked to the fact that this gene also has been described as being involved in weight gain induced by antipsychotic drugs [5, 6, 10]. It is possible that, even in patients that have not yet shown weight gain, leptin resistance has developed. Whether or not this is the case warrants further investigation.

The presence of the T allele of rs17782313 SNP of the* MC4R* gene was associated with higher HOMA-IR index levels. The MC4R receptor, encoded by the* MC4R* gene, is important in the leptin pathway although it is not directly involved [12]. The findings of our study suggest that the* MC4R* gene may indeed play a role in leptin modulation. Although in this study the* MC4R* gene was not associated with BMI, Willer et al. [27] performed a meta-analysis of 15 genome-wide association studies (GWAS) for BMI, comprising 32,387 participants and followed up top signals in 14 additional cohorts comprising 59,082 participants [27]. They strongly confirmed previous reports of association with* MC4R* at SNP rs17782313 with a per-allele change in BMI of 0.20 and an overall *p* value of 1.1 × 10^−20^ [27].

The SNP rs7799039 of the* LEP* gene, when the A allele was absent, was associated with a higher frequency of obesity. The presence of the G allele was associated with higher levels of ALT, which is a common finding in obesity-related hepatic steatosis [28]. This finding agrees with the results found by Templeman et al. [29] but differs from the negative findings from Fernández et al. [30] and the opposite result found by Wu et al. [31], in which studies weight gain was described in those patients with the A allele. According to Shen et al., these contradictory findings may be due to ethnic differences, with the A allele being a risk factor in Asian populations but a protective factor in European populations [32]. The absence of the A allele of SNP rs1137101 from the* LEPR* gene, which encodes the leptin receptor, was associated with higher serum concentrations of leptin. In the present study, it was found most elevated in homozygous A/A subjects.

Considering the* CYP2D6* gene, the absence of the C allele (T/T genotype) was associated with increased weight and the occurrence of hypertension. Homozygosity (C/C and T/T) was associated with a higher HOMA-IR, and the presence of the C allele was associated with higher ALT values. Indeed,* CYP2D6* is currently the only gene included in the pharmacogenomic biomarker frame labeling developed by the FDA for risperidone, with attention being given to poor metabolizers [33].

In contrast to the findings of this study, Melkersson et al. have reported that the* CYP2D6* gene is not directly associated with a greater frequency of hypertension and values of HOMA-IR index change [34]. However, our findings do agree with those of Lane et al., in which fewer patients using risperidone were C/C in genotype than those that were C/T or T/T [35]. Correia et al. have also reported that associations between the* CYP2D6* gene and weight gain are most pronounced among poor risperidone metabolizers [14].

Many SNP variants of the* DRD2* gene have been associated with weight gain induced by antipsychotics [36, 37]. In this study, there were no specific associations of the two researched* DRD2* SNPs with weight gain, but both were associated with HOMA IR. To the best of the authors' knowledge, this association has not been previously reported.

Among the biochemical and hormonal laboratory parameters investigated, a gender difference was only observed for leptin concentrations and AST values, which are physiologically expected to be higher and lower, respectively, in females. In boys, leptin concentrations decrease and AST increases during puberty due to a greater gain of lean mass over fat. For females, the opposite occurs because girls gain body fat during puberty, resulting in increased leptin concentrations [38].

Thirty-seven (30.9%) children and adolescents were overweight or obese, a higher proportion than the general Brazilian population in the same age group, where 21.7% of male adolescents and 19.4% of females are overweight [39]. At present, obesity is viewed as a pandemic that accounts for about three million deaths annually worldwide [40].

Metabolic syndrome was found in six (5%) subjects: proportionally slightly higher than that reported in the Third National Health and Nutrition Examination Survey (NHANES III) which recorded metabolic syndrome in 4.2% of adolescents aged 12–19 years [41] and 9.2% assessed by a study that also used data from adolescents form NHANES III but with another cut-off [42]. While Cook et al. (2003) adopted the parameters established by the National Cholesterol Education Program Adult Treatment Panel III (NCEP/ATP III) [21, 41]; the other adapted the definition according to age [21, 42]. In the present study, we used the criteria defined by IDF [23]. Tavares-Giannini et al. (2014) conducted a study of obese or overweight adolescents only (*n* = 232) from a public school in Rio de Janeiro [43] and compared both criteria. They found metabolic syndrome in 40.4% and 24.6% of obese adolescents and 9.4% and 1.9% of overweight adolescents using the NCEP/ATPIII and IDF criteria, respectively [21, 23, 43]. These findings show that the IDF criteria, as a result of being more restrictive, may not consider the totality of children and adolescents at increased risk for developing diabetes and cardiovascular disease [43]. Nevertheless, in this study, occurrence was greater than in the study of Tavares-Giannini et al. where the same criteria were used in a population with similar characteristics, except for the use of risperidone [43].

A high frequency of dyslipidemia with both high levels of total cholesterol and triglyceride was found, which is in accordance with the literature [42, 44]. The occurrence of dyslipidemia in this sample was higher, confirming the potential influence of risperidone in such changes [42]. Besides weight gain, child and adolescent risperidone users seem more sensitive than adults to adverse metabolic effects, particularly lipid abnormalities [45].

No patient was found to have glucose levels above 126 mg/dL. Lower rates of diabetes than those found in adults taking antipsychotics is easily explained by the shorter follow-up time of young people continuously using the medication, and by the fact that diabetes mellitus and cardiovascular complications are correlated with age and the duration of the presence of cardiovascular risk factors [45]. The HOMA-IR index was abnormal in 18.3% of the patients, and this is in agreement with other studies that have reported increased HOMA-IR among children and adolescents using risperidone [41]. This index can reflect not only early pancreatic damage, since the product is composed of glucose and insulin, but also liver damage that may be caused by excess lipids and evaluated using ALT levels [20].

Waist circumference (WC), which was increased in 16.7% of the patients, is a good early indicator of metabolic disorders. It has also been reported, even after adjusting for age, to be significantly higher in children with abnormal glucose metabolism, hyperinsulinemia, and high HOMA-IR [46]. WC is a simple and sensitive way to determine the risk of developing metabolic syndrome in children treated with second-generation antipsychotics [44].

WC and blood pressure and systolic and diastolic levels were positively correlated with age. AST values, as expected [47], correlated negatively with age. This is in accordance with the physiological increase expected with the growth foreseen in anthropometric age curves for these measurements [18, 21].

Higher leptin levels found in children with higher doses of risperidone per kilogram of body weight may suggest that these children are in the early stages of developing leptin intolerance. Higher HOMA-IR values in patients with increased bone age point to a potential risk of developing type 2 diabetes mellitus.

Among the limitations of this study, the fact that it was a transversal study did not allow access to clinical and laboratory parameters before treatment, so it was unable to establish causal relationships. However, the design of the SNP portion of the study was not inappropriate since genes determine predisposition.

Studies of candidate gene analysis are important for advances in pharmacogenetics. Nevertheless, newer techniques for analyzing the human genome have accelerated greatly the knowledge on this field [13]. These include genome-wide association studies and the so-called next-generation sequencing methods, which refer to the whole genome and to the whole exome sequencing [13]. These techniques are still less available and more expensive, but they enable precise, fast, and effective research of multiple possible SNPs underpinning traits and drug responses or adverse effects [13]. However, despite all these advantages, it should be considered that, in none of these methods, the concordance between genotype and phenotype is absolute [13]. The phenotypes can also be influenced by conditions such as drug-drug interactions, age, sex, and comorbid conditions [13].

Because of logistical limitations, this study was unable to collect data such as metabolic rate, calorie intake, and physical activity as well as the serum levels of risperidone and its active metabolite, 9-hydroxy-risperidone. It is worth noting that none of the studies reviewed here have assessed these variables, making them worthy of future investigation.

Although many of the patients in this study were not on monotherapy with risperidone and several other psychiatric drugs can influence weight gain and metabolic complications, more than a quarter of the sample did not use other drugs, and there was no difference in the prevalence of obesity, altered AC, hypertension, and metabolic syndrome between groups.

## 5. Conclusion

In summary, the results show that a high frequency of child and adolescent users of risperidone have metabolic disorders and/or are overweight, despite being given therapeutic doses considered to be suitable for their age. There were correlations between age and risperidone dose with AC, SBP, and triglyceride concentrations and between patients' age and DBP, insulin, AST, and leptin levels.

In the analyzed sample, it is clear that many children and adolescents do not use risperidone as a monotherapy which increases the risk of adverse effects. Some SNPs analyzed were associated with adverse effects of risperidone. The results highlight the importance of clinical, metabolic, and genetic factors in adverse responses to a medication widely used in child and adolescent psychiatry. Future pharmacogenetic studies will help further identify risk factors and facilitate advances in individualizing antipsychotic treatment and the development of therapeutic tools with better safety and tolerability profiles.

## Figures and Tables

**Table 1:** Frequency of clinical or laboratory abnormalities in the 120 child and adolescent users of risperidone assessed by the study.

Abnormalities	*n* (%)
Overweight	32 (26.7)
Obese	5 (4.2)
Diabetes mellitus	0
BP^*∗*^	8 (6.7)
Metabolic syndrome	6 (5)
Increased waist circumference^§^	20 (16.7)
HOMA-IR changes	22 (18.3)
Total hypercholesterolemia	32 (26.7)
Hypercholesterolemia	29 (24.2)
Hypocholesterolemia	40 (33.3)
Hypertriglyceridemia	41 (34.2)

BP, blood pressure; HOMA-IR, homeostatic model assessment of insulin resistance.

^*∗*^16 (13.3%) subjects were classified as prehypertensive; ^§^44 (36.7%) subjects were classified as being between the 75th and 90th percentiles for waist circumference.

**Table 2:** Mean values and SD of clinical and laboratory parameters in relation to total assessments and nutritional status.

Parameter	Total	Eutrophic	Overweight	Obese	*p* ^#^
		(*n* = 83)	(*n* = 32)	(*n* = 5)	
Prescribed dose^%^ (mg/day)	2.1 ± 1.3	2.0 ± 1.3	2.4 ± 1.3	2.1 ± 1.4	0.250
Adjusted dose^%%^ (mg/Kg)	0.04 ± 0.03	0.05 ± 0.03	0.04 ± 0.02	0.03 ± 0.02	0.343
Time used (months)	25.9 ± 27.2	24.7 ± 27.6	29.0 ± 27.0	27.4 ± 24.9	0.369
Age (years)	13.0 ± 3.1	12.9 ± 2.9	13.5 ± 3.6	10.7 ± 3.1	0.176
Bone age (years)	13.0 ± 3.1	13.0 ± 3.1	13.3 ± 3.2	10.9 ± 3.0	0.305
WC (cm)	75.4 ± 11.5	70.8 ± 9.3	86 ± 9.0^§^	84.5 ± 9.2^*∗*^	**<0.001**
SBP (mmHg)	102.5 ± 11.8	100.6 ± 10.3	106.6 ± 12.6^§^	108 ± 21.7	0.080
DBP (mmHg)	63.5 ± 11.3	62.1 ± 11.1	67.5 ± 10.8^§^	62 ± 14.8	0.078
Glycemia (mg/mL)	85.2 ± 8.5	84.2 ± 7.7	86.7 ± 8.5	93.6 ± 15.0	0.120
Insulin (nUI/mL)	9.4 ± 9.8	7.5 ± 8.3	14.2 ± 12.0^§^	10.1 ± 8.1	**0.003**
HOMA-IR index	2.0 ± 2.3	1.6 ± 2.1	3.0 ± 2.5^§^	2.5 ± 2.0	**0.002**
Total cholesterol (mg/dL)	156.2 ± 35.9	151.4 ± 31.2	164.0 ± 43.7	184.6 ± 40.7	0.096
LDL cholesterol (mg/dL)	87.5 ± 29.1	82.8 ± 26.2	96.4 ± 33.2^§^	107.0 ± 31.1	**0.045**
HDL cholesterol (mg/dL)	52.7 ± 14.1	53.9 ± 14.9	48.7 ± 12.0	57.4 ± 5.9	0.119
Triglycerides (mg/dL)	80.3 ± 45.4	73.9 ± 38.6	93.5 ± 56.8	101.0 ± 55.7	0.081
AST (U/L)	23.4 ± 10.0	23.4 ± 10.9	22.8 ± 6.7	28.2 ± 12.4	0.475
ALT (U/L)	18.4 ± 16.9	16.5 ± 17.0	21.1 ± 11.8^§^	31.4 ± 33.0	**0.001**
Leptin (pg/mL)	26.0 ± 16.4	19.2 ± 12.7	40.8 ± 13.6^§^	44.3 ± 8.5^*∗*^	**<0.001**

WC, waist circumference; SBP, systolic blood pressure; DBP, diastolic blood pressure.

^#^Kruskal-Wallis test.

^§^Statistically significant difference (by Mann-Whitney test) between overweight and normal weight.

^*∗*^Statistically significant difference (by Mann-Whitney test) between obese and normal weight individuals (*p* = 0.05).

^%^Total prescribed risperidone.

^%%^Prescribed dose of risperidone, but adjusted per kg of body weight.

**Table 3:** Values of Spearman correlation test and significance level (*p*) between clinical and laboratory parameters with age, duration of use, and dose of risperidone in the study sample.

Parameters	ChronologicAge (years)	Bone age (years)	Length of risperidone use (months)	Risperidone dose (mg)	Dose (mg) per kilogram of body weight
BMI	−0.015 (0.872)	0.017 (0.851)	0.026 (0.781)	0.090 (0.327)	−0.217 (0.017)

WC (cm)	**0.395** **(0.0001)**	**0.409** **(0.0001)**	0.030 (0.746)	**0.217** **(0.017)**	−0.151 (0.100)

SBP (mmHg)	**0.487** **(0.0001)**	**0.501** **(0.0001)**	0.202 (0.027)	**0.203** **(0.027)**	−0.080 (0.386)

DBP (mmHg)	**0.335** **(0.0001)**	**0.308** **(0.001**)	0.176 (0.055)	0.126 (0.169)	−0.041 (0.658)

Total cholesterol (mg/dL)	−0.008 (0.928)	−0.051 (0.580)	−0.068 (0.462)	0.119 (0.194)	0.075 (0.415)

LDL cholesterol (mg/dL)	0.057 (0.537)	−0.007 (0.940)	−0.037 (0.687)	0.075 (0.414)	0.008 (0.932)

HDL cholesterol (mg/dL)	−0.171 (0.062)	**−0.183** **(0.046)**	−0.138 (0.133)	0.024 (0.791)	0.120 (0.193)

Triglycerides (mg/dL)	0.126 (0.170)	0.147 (0.108)	−0.023 (0.806)	0.115 (0.213)	−0.25 (0.784)

HOMA-IR index	0.169 (0.065)	**0.185** **(0.043)**	0.021 (0.818)	0.088 (0.340)	−0.092 (0.320)

AST (U/L)	**−0.322** **(0.0001)**	**−0.283** **(0.002)**	0.009 (0.925)	−0.070 (0.450)	0.043 (0.644)

ALT (U/L)	0.019 (0.840)	0.017 (0.858)	−0.023 (0.801)	0.100 (0.277)	−0.013 (0.889)

Leptin (pg/mL)	0.113 (0.217)	0.087 (0.345)	−0.045 (0.624)	−0.012 (0.898)	**−0.257** **(0.005)**

WC, waist circumference; SBP, systolic blood pressure; DBP, diastolic blood pressure.

**Table 4:** Absolute and relative frequencies (%) of alleles of SNPs for the presence or absence of clinical and laboratory changes evaluated as discrete variables (*n* = 120).

	Genotype			*χ* ^2^	*p*
Obese	*LEP*				
	AA	AG	GG		

Yes	—	—	**5 (10.9)**	8.393	0.015^§§^
No	16 (100)	58 (100)	41 (89.1)		

Obese	*LEP*				
	Presence of allele A		Absence of allele A		

Yes	—		**5 (10.9)**	8.393	0.007^*∗*^
No	74 (100)		41 (89.1)		

Obese or overweight	*CYP2D6*				
	CC	CT	TT		

Yes	25 (32.5)	7 (19.4)	**5 (71.4)**	7.695	0.021^§§^
No	52 (67.5)	29 (80.6)	2 (28.6)		

Obese or overweight	*CYP2D6*				
	Presence of allele C		Absence of allele C		

Yes	32 (28.3)		**5 (71.4)**	5.744	0.028^*∗*^
No	81 (71.7)		2 (28.6)		

Obese or overweight	*HTR2C* rs3813929 (Both genders)				
	Presence of allele G		Absence of allele G		

Yes	**33 (35.1)**		4 (15.4)	3.714	0.054^§^
No	61 (64.9)		22 (84.6)		

Obese or overweight	Females				
	Presence of allele G		Absence of allele G		

Yes	8 (47.1)		—	3.697	0.115^*∗*^
No	9 (52.9)		5 (100)		

Obese or overweight	Males				
	Presence of allele G		Absence of allele G		

Yes	25 (32.5)		4 (19)	1.426	0.289^§^
No	52 (67.5)		17 (81)		

HOMA-IR	*DRD2* rs1799978				
	AA	AG	GG		

Changed	12 (13.2)	**10 (34.5)**	—	6.661	0.010^§§^
Normal	79 (86.8)	19 (65.5)	—		

HOMA-IR	*DRD2* rs6277				
	Presence of allele T		Absence of allele T		

Changed	6 (10.9)		**16 (24.6)**	3.738	0.053^§^
Normal	49 (89.1)		49 (75.4)		

Hypertension	*CYP2D6*				
	CC	CT	TT		

Yes	3 (3.9)	3 (8.3)	**2 (28.6)**	6.509	0.039^§§^
No	74 (96.1)	33 (91.7)	5 (71.4)		

Abdominal circumference	*HTR2C* rs6318 (both genders)				
	C, CC	CG	G, GG		

Increased	1 (3.8)	**3 (42.9)**	16 (18.4)	6.720	0.035^§§^
Normal	25 (96.2)	4 (57.1)	71 (81.6)		

	Females				
	CC	CG	GG		

Increased	—	**2 (40)**	—	7.480	0.024^§§^
Normal	5 (100)	3 (60)	12 (100)		

	Males				
	C		G		

Increased	1 (4.5)		17 (22.4)	3.615	0.066^*∗*^
Normal	21 (95.5)		59 (77.6)		

^§^Chi-square (GL = 1); ^§§^Chi-square (GL = 2); ^*∗*^Fisher's exact test.

**Table 5:** Average rank (Mann-Whitney test) of continuous variables evaluated in relation to the SNP alleles (*n* = 120).

SNP	Parameter	*n*	Average Rank	*p*
	Leptin			

*HTR2C* rs3813929 (both genders)	Hemi/homozygous	115	59.17	**0.044**
	Heterozygotes	5	**91.20**	

*HTR2C* rs3813929 (females)	Heterozygotes	5	9.10	0.704
	Homozygous	17	12.21	

*HTR2C* rs3813929 (males)	Hemizygous C	85	48.16	0.232
	Hemizygous T	13	58.27	

*LEPR*	Presence of allele A	93	57.04	**0.043**
	Absence of allele A	27	**72.41**	

	ALT			

*LEP*	Absence of allele G	16	44.84	**0.053**
	Presence of allele G	104	**62.91**	

*CYP2D6*	Absence of allele C	113	58.60	**0.016**
	Presence of allele C	7	**91.14**	

	HOMA-IR			

*MC4R*	Absence of allele T	7	35.14	**0.047**
	Presence of allele T	113	**62.07**	

*CYP2D6*	Heterozygotes	36	56.38	**0.032**
	Homozygous	84	**62.27**	

## Abbreviations

SNP: Single nucleotide polymorphisms
HTR2C: Serotonin 2C receptor
DRD2: Dopamine receptor D2
LEP: Leptin
LEPR: Leptin receptor
MC4R: Melanocortin 4 receptor
CYP2D6: Cytochrome P450, family 2, subfamily D, polypeptide 6
WC: Waist circumference
BMI: Body mass index
BP: Blood pressure
HOMA-IR: Homeostatic model assessment of insulin resistance index
GWAS: Genome-wide association study.

## References

[1] Cabaleiro T., Ochoa D., López-Rodríguez R. (2014). Effect of polymorphisms on the pharmacokinetics, pharmacodynamics, and safety of risperidone in healthy volunteers. *Human Psychopharmacology*.

[2] Stahl S. (2008). *Stahl's Essential Psychopharmacology: Neuroscientific Basis and Practical Applications*.

[3] FDA (2007). *Drug Approved for Two Psychiatric Conditions in Children and Adolescents*.

[4] McKinney C., Renk K. (2011). Atypical antipsychotic medications in the management of disruptive behaviors in children: safety guidelines and recommendations. *Clinical Psychology Review*.

[5] Lett T. A. P., Wallace T. J. M., Chowdhury N. I., Tiwari A. K., Kennedy J. L., Müller D. J. (2012). Pharmacogenetics of antipsychotic-induced weight gain: review and clinical implications. *Molecular Psychiatry*.

[6] Reynolds G. P. (2012). Pharmacogenetic aspects of antipsychotic drug-induced weight gain—a critical review. *Clinical Psychopharmacology and Neuroscience*.

[7] Hoekstra P. J., Troost P. W., Lahuis B. E. (2010). Risperidone-induced weight gain in referred children with autism spectrum disorders is associated with a common polymorphism in the 5-hydroxytryptamine 2C receptor gene. *Journal of Child and Adolescent Psychopharmacology*.

[8] Müller D. J., Zai C. C., Sicard M. (2012). Systematic analysis of dopamine receptor genes (DRD1-DRD5) in antipsychotic-induced weight gain. *Pharmacogenomics Journal*.

[9] Wang G.-J., Volkow N. D., Logan J. (2001). Brain dopamine and obesity. *The Lancet*.

[10] Perez-Iglesias R., Mata I., Amado J. A. (2010). Effect of FTO, SH2B1, LEP, and LEPR polymorphisms on weight gain associated with antipsychotic treatment. *Journal of Clinical Psychopharmacology*.

[11] Grunberger G., Garber A., Mechanick J. (2014). Obesity management: applying clinical trial data to clinical care. *Endocrine Practice*.

[12] List J. F., Habener J. F. (2003). Defective melanocortin 4 receptors in hyperphagia and morbid obesity. *The New England Journal of Medicine*.

[13] Maggo S. D. S., Savage R. L., Kennedy M. A. (2015). Impact of new genomic technologies on understanding adverse drug reactions. *Clinical Pharmacokinetics*.

[14] Correia C. T., Almeida J. P., Santos P. E. (2010). Pharmacogenetics of risperidone therapy in autism: association analysis of eight candidate genes with drug efficacy and adverse drug reactions. *Pharmacogenomics Journal*.

[15] Tanner J., Whitehouse R. (1975). *Assessment of Skeletal Maturity and Prediction of Adult Height (TW2 Method)*.

[16] WHO—World Health Organization (2007). *Growth Reference 5–19 Years*.

[17] Must A., Anderson S. E. (2006). Body mass index in children and adolescents: considerations for population-based applications. *International Journal of Obesity*.

[18] Fernández J. R., Redden D. T., Pietrobelli A., Allison D. B. (2004). Waist circumference percentiles in nationally representative samples of African-American, European-American, and Mexican-American children and adolescents. *Journal of Pediatrics*.

[19] National High Blood Pressure Education Program Working Group on High Blood Pressure in Children and Adolescents (2004). The fourth report on the diagnosis, evaluation, and treatment of high blood pressure in children and adolescents. *Pediatrics*.

[20] Aradillas-García C., Rodríguez-Morán M., Garay-Sevilla M. E., Malacara J. M., Rascon-Pacheco R. A., Guerrero-Romero F. (2012). Distribution of the homeostasis model assessment of insulin resistance in Mexican children and adolescents. *European Journal of Endocrinology*.

[21] De Jesus J. M. (2011). Expert panel on integrated guidelines for cardiovascular health and risk reduction in children and adolescents: summary report. *Pediatrics*.

[22] World Health Organization (1999). *Definition, Diagnosis and Classification of Diabetes Mellitus and Its Complications. Part 1: Diagnosis and Classification of Diabetes Mellitus*.

[23] IDF International Diabetes Federation The definition of metabolic syndrome in children. http://www.idf.org/webdata/docs/Mets_definition_children.pdf.

[24] Pena S. D. J., Bastos-Rodrigues L., Pimenta J. R., Bydlowski S. P. (2009). DNA tests probe the genomic ancestry of Brazilians. *Brazilian Journal of Medical and Biological Research*.

[25] Ministério da Educação (2005). *Mostre Sua Raça, Declare Sua Cor*.

[26] McCarthy S., Mottagui-Tabar S., Mizuno Y. (2005). Complex HTR2C linkage disequilibrium and promoter associations with body mass index and serum leptin. *Human Genetics*.

[27] Willer C. J., Speliotes E. K., Loos R. J. F. (2009). Six new loci associated with body mass index highlight a neuronal influence on body weight regulation. *Nature Genetics*.

[28] Botros M., Sikaris K. A. (2013). The de ritis ratio: the test of time. *Clinical Biochemist Reviews*.

[29] Templeman L. A., Reynolds G. P., Arranz B., San L. (2005). Polymorphisms of the 5-HT2C receptor and leptin genes are associated with antipsychotic drug-induced weight gain in Caucasian subjects with a first-episode psychosis. *Pharmacogenetics and Genomics*.

[30] Fernández E., Carrizo E., Fernández V. (2010). Polymorphisms of the *LEP*- and *LEPR* genes, metabolic profile after prolonged clozapine administration and response to the antidiabetic metformin. *Schizophrenia Research*.

[31] Wu R., Zhao J., Shao P., Ou J., Chang M. (2011). Genetic predictors of antipsychotic-induced weight gain: a case-matched multi-gene study. *Zhong Nan Da Xue Xue Bao Yi Xue Ban*.

[32] Shen J., Ge W., Zhang J., Zhu H. J., Fang Y. (2014). Leptin −2548g/a gene polymorphism in association with antipsychotic-induced weight gain: a meta-analysis study. *Psychiatria Danubina*.

[33] U.S. Food and Drug Administration Table of pharmacogenomic biomarkers in drug labels. http://www.fda.gov/drugs/scienceresearch/researchareas/pharmacogenetics/ucm083378.Htm.

[34] Melkersson K. I., Scordo M. G., Gunes A., Dahl M.-L. (2007). Impact of CYP1A2 and CYP2D6 polymorphisms on drug metabolism and on insulin and lipid elevations and insulin resistance in clozapine-treated patients. *Journal of Clinical Psychiatry*.

[35] Lane H.-Y., Liu Y.-C., Huang C.-L. (2006). Risperidone-related weight gain: genetic and nongenetic predictors. *Journal of Clinical Psychopharmacology*.

[36] Wallace T. J., Zai C. C., Brandl E. J., Müller D. J. (2011). Role of 5-HT_2C_ receptor gene variants in antipsychotic-induced weight gain. *Pharmacogenomics and Personalized Medicine*.

[37] Zhang J.-P., Malhotra A. K. (2011). Pharmacogenetics and antipsychotics: therapeutic efficacy and side effects prediction. *Expert Opinion on Drug Metabolism and Toxicology*.

[38] Meira T. B., de Moraes F. L., Böhme M. T. S. (2009). Relações entre leptina, puberdade e exercício no sexo feminino. *Revista Brasileira de Medicina do Esporte*.

[39] IBGE—Instituto Brasileiro de Geografia e Estatística (2010). Pesquisa de orçamentos familiares 2008-2009. *Antropometria e Estado Nutricional de Crianças, Adolescentes e Adultos no Brasil*.

[40] Finucane M. M., Stevens G. A., Cowan M. J. (2011). National, regional, and global trends in body-mass index since 1980: systematic analysis of health examination surveys and epidemiological studies with 960 country-years and 9·1 million participants. *The Lancet*.

[41] Cook S., Weitzman M., Auinger P., Nguyen M., Dietz W. H. (2003). Prevalence of a metabolic syndrome phenotype in adolescents: findings from the Third National Health and Nutrition Examination Survey, 1988–1994. *Archives of Pediatrics and Adolescent Medicine*.

[42] De Ferranti S. D., Gauvreau K., Ludwig D. S., Neufeld E. J., Newburger J. W., Rifai N. (2004). Prevalence of the metabolic syndrome in American adolescents: findings from the Third National Health and Nutrition Examination Survey. *Circulation*.

[43] Tavares-Giannini D., Caetano-Kuschnir M. C., Szklo M. (2014). Metabolic syndrome in overweight and obese adolescents: a comparison of two different diagnostic criteria. *Annals of Nutrition and Metabolism*.

[44] Panagiotopoulos C., Ronsley R., Kuzeljevic B., Davidson J. (2012). Waist circumference is a sensitive screening tool for assessment of metabolic syndrome risk in children treated with second-generation antipsychotics. *Canadian Journal of Psychiatry*.

[45] Maayan L., Correll C. U. (2011). Weight gain and metabolic risks associated with antipsychotic medications in children and adolescents. *Journal of Child and Adolescent Psychopharmacology*.

[46] Calarge C. A., Nicol G., Schlechte J. A., Burns T. L. (2014). Cardiometabolic outcomes in children and adolescents following discontinuation of long-term risperidone treatment. *Journal of Child and Adolescent Psychopharmacology*.

[47] Motta V. (2009). *Clinical Biochemistry for Laboratory—Principles and Interpretations*.

