# Corrigendum to “Pharmacogenetics of Risperidone and Cardiovascular Risk in Children and Adolescents”

**DOI:** 10.1155/2017/8165368

**Published:** 2017-05-03

**Authors:** Amilton Dos Santos-Júnior, Taciane Barbosa Henriques, Maricilda Palandi de Mello, Osmar Henrique Della Torre, Lúcia Arisaka Paes, Adriana Perez Ferreira-Neto, Letícia Esposito Sewaybricker, Thiago Salum Fontana, Eloisa Helena Rubello Valler Celeri, Gil Guerra-Júnior, Paulo Dalgalarrondo

**Affiliations:** ^1^Department of Psychiatry, School of Medical Sciences (FCM), State University of Campinas (Unicamp), 13083-887 Campinas, SP, Brazil; ^2^Laboratory of Human Genetics, Center for Molecular Biology and Genetic Engineering (CBMEG), Unicamp, 13083-875 Campinas, SP, Brazil; ^3^Growth and Development Laboratory, Center for Investigation in Pediatrics (CIPED), FCM-Unicamp, 13083-887 Campinas, SP, Brazil; ^4^Department of Pediatrics, Pediatric Endocrinology Unit, FCM-Unicamp, 13083-887 Campinas, SP, Brazil

In the article titled “Pharmacogenetics of Risperidone and Cardiovascular Risk in Children and Adolescents” [1], there was an error in the “Laboratory Evaluation” section, where the selected CYP2D6 SNP is written as rs72552269, and should be corrected to “rs1065852.”
